# Efficacy of the Whole-Body Cryotherapy as Add-on Therapy to Pharmacological Treatment of Depression—A Randomized Controlled Trial

**DOI:** 10.3389/fpsyt.2020.00522

**Published:** 2020-06-09

**Authors:** Joanna Rymaszewska, Katarzyna M. Lion, Lilla Pawlik-Sobecka, Tomasz Pawłowski, Dorota Szcześniak, Elżbieta Trypka, Julia E. Rymaszewska, Agnieszka Zabłocka, Bartlomiej Stanczykiewicz

**Affiliations:** ^1^Department of Psychiatry, Wroclaw Medical University, Wroclaw, Poland; ^2^Department of Nervous System Diseases, Wroclaw Medical University, Wroclaw, Poland; ^3^Student Scientific Association at Department of Psychiatry, Wroclaw Medical University, Wroclaw, Poland; ^4^Laboratory of Microbiome Immunobiology, Hirszfeld Institute of Immunology and Experimental Therapy, Polish Academy of Sciences, Wroclaw, Poland

**Keywords:** whole-body cryotherapy, cryostimulation, depression, non-pharmacological treatment, mood disorder’, mental health

## Abstract

**Introduction:**

Accumulating evidence indicates the effectiveness of cryogenic temperature interventions in rheumatoid arthritis, ankylosing spondylitis, fibromyalgia, multiple sclerosis, and chronic low back pain. The application of whole-body cryotherapy (WBC) in psychiatric aspects of medicine was also noted. Nevertheless, the exact mechanisms explaining the beneficial effect of WBC on mood disorders remain unclear. The study aimed to assess the efficacy of repetitive short exposure to extremely low temperatures (WBC) on mood, quality of life as well as on biochemical measures among people diagnosed with depressive episode undergoing pharmacological treatment.

**Materials and Methods:**

Prospective randomized, double-blind sham-controlled protocol was used. The study enrolled 92 medically stable adults (aged 20–73 years) with a diagnosis of a depressive episode. The participants were randomly allocated and exposed to 10 whole-body cryotherapy (WBC) sessions (−110°C till −160°C [the experimental group (EG)] or to low, but not cryogenic temperatures −50°C [the control group (CG)]. Thirty participants in the EG and 26 in CG completed the whole study. The primary outcome measures were depressive symptoms evaluated with the Beck Depression Inventory-II (BDI-II) as well as the Hamilton Depression Rating Scale (HAM-D 17). The quality of life, quality of sexual life, acceptance of the disease and self-reported mood, vitality, and sleep quality were assessed as secondary outcome measures. The study was registered at Australian New Zealand Clinical Trials Registry (ACTRN12619001600134).

**Results:**

The results show evidence for a statistically significant difference in the clinical assessment of depressive symptoms according to HAM-D 17 scale (T4 by group interaction p=0.02), BDI-II (T2 time by group interaction p=0.01), cognitive-affective BDI dimension (T4 by group interaction p=0.00), and somatic BDI dimension (T4 by group interaction p=0.028). Significant improvement was also noticed in life quality (p < 0.05), self-assessed mood (p=0.035), and disease acceptance (p=0.007). There were no statistically significant changes related to sexual satisfaction, self-assessed vitality, and sleep (p > 0.05).

**Conclusions:**

Whole-body cryotherapy is a useful method to improve standard pharmacological treatment. The WBC intervention reduces mental health deterioration, especially in mood disorders, such as depression, and can be beneficial for well-being and quality of life.

## Introduction

Mood disorders are one of the major causes of human suffering in the world and are one of the most significant challenges for modern medicine (1). Currently, over 322 million people are diagnosed with depression (2), and this number is still rising. The World Health Organization predicts that depression and ischemic heart disease will become two major reasons for disabilities in the world by 2020 (2–4). Among the 25 most common causes of global disability-adjusted life-years (DALYs), for both sexes combined, depressive disorders change the position from 15 in 1990, to 14 in 2005 to 11 in 2013 (1). More importantly, depression influences the course and prognosis of many somatic disorders, including ischemic heart disease, diabetes, or obesity. It is also a cause of death of about 1 million worldwide annually (5).

Antidepressants are routinely used for the treatment of depression worldwide. Recently, the meta-analysis published by Cipriani et al. (6) revealed that all 21 anti-depressants were more efficacious than placebo in adults with major depressive disorder. Especially, escitalopram, mirtazapine, paroxetine, agomelatine, and sertraline had a relatively higher response and lower dropout rate than the other antidepressant (6). Nevertheless, in a STAR*D survey, only 27% of patients met the remission criteria [measured in the Hamilton Depression Rating Scale (HAM-D 17) scale] after the first stage of treatment (with citalopram). However, the accumulated remission ratio after the complete four stages of treatment equaled 67% (7). Several additional medication strategies are used to potentiate antidepressant treatment. The augmentation approach may include antipsychotics, mood stabilizers, anxiolytics, lithium, thyroid hormone, or other drugs. Currently, many new treatment solutions for depressive patients are developed and studied, such as repetitive transcranial magnetic stimulation (rTMS) (8), intravenous/intranasal ketamine (9), inhaled nitrous oxide (10), valgus nerve stimulation (11), deep brain stimulation (12), buprenorphine (13), etc.

The whole-body cryotherapy (WBC) is a short (1–3 minutes), repetitive exposition to extremely low temperatures in specially designed cryochambers, where the temperature reaches from −110° to −160° Celsius degrees.

The effectiveness of cryogenic temperature treatments is proven in the treatment and rehabilitation of several diseases like multiple sclerosis, arthrosis, chronic back pain, or fibromyalgia (14–17). We also know that WBC is widely used in sports medicine (18) and may have a potentially positive effect on affective disorders (19–21), deterioration of cognitive functions (22), and worsen sleep quality (23, 24) as well. The potential mechanisms of action still remain unclear. However, the influence of WBC *via* alleviation of an inflammatory process or reduction of oxidative stress has been reported [(25, 26), for review see: (14)].

Potentially, WBC, if used as an additional method of pharmacotherapy in depression, might affect hormonal, immune, and anti-inflammatory changes. Particularly, inflammation is considered as one of the depression causes or pathomechanism (27, 28). Meta-analyses have shown that inflammation is connected with depression, i.e., the elevated C-reactive protein, interleukin 6 (IL-6), and tumor necrosis factor alpha (TNF-α) in major depressive disorder (29, 30). The study of Pawlowski et al. revealed that activation of peripheral blood mononuclear cells (PBMC), as measured by the level of cytokine and chemokine transcripts, correlates with depression and neuroticism scores (31). Additionally, a systematic review and meta-analysis performed by Black et al. (32) confirmed that oxidative stress markers are increased in subjects with depression. Moreover, a decreased level of nitric oxide (NO) in depressive patients was found (33, 34).

It was reported that WBC upregulates the activity of the immune system by increasing the level of IL-6 and IL-10 cytokines and decreasing of IL-1α and total antioxidant status (TAS) in healthy people (35). However, the significant reduction of CRP, IL-1β, TNFα, and also IL-6 was determined in obese men or people with ankylosing spondylitis after cryostimulation (26, 36, 37). It was also observed that the level of oxidative stress markers decreased after the exposition to WBC linked with the physical exercises (38), and what is more, the obtained antioxidant effect does not depend on the type of cryochambers in which the WBC procedures were performed (25). These observations suggest that WBC can act as modulatory upregulation of the immune response of healthy people and also as downregulation of the inflammatory response in pathological conditions (26, 36, 37). Based on this, the potential influence of WBC on the inflammatory process, also related to depression, is considered.

The study aimed to assess the efficacy of repetitive short exposure to extremely low temperatures (WBC) on mood, quality of life as well as biochemical measures among people diagnosed with depressive episode undergoing pharmacological treatment.

## Materials and Methods

### Participants

The project was based on prospective randomized, double-blind sham-controlled protocol and was registered at the Australian New Zealand Clinical Trials Registry (ANZCTR) (ACTRN12619001600134). All participants received oral and written information about the study protocol, anonymity, possibility to resign at any time of the study, and gave the written consent.

The study enrolled 92 medically stable (based on physical examination, medical history, and vital signs at screening) adults (aged 20–73 years) with a diagnosis of a depressive episode [F.32 or F.33 according to research criteria of International Classification of Diseases (ICD-10)]. All participants were treated in outpatient clinics. After enrolment, there were no changes in dosage or type of antidepressants [selective serotonin reuptake inhibitor (SSRI) or serotonin-norepinephrine reuptake inhibitor (SNRI)] during the study.

The exclusion criteria were alcohol and drug abuse, dementia diagnosis, inability to understand questions and written information, psychosis, suicidal thoughts, standard contraindications to use WBC (e.g., acute respiratory diseases, acute cardiovascular disease like coronary disease, circulatory insufficiency, unstable hypertension, cold intolerance, claustrophobia, cryoglobulinemia, cancer, deep vein diseases, hypothyroidism, neuropathies, purulent skin changes, Reynaud disease, pregnancy), and previous exposition to WBC treatment.

Before starting the WBC sessions, 34 people resigned from the study (declaring lack of time, claustrophobia, fear of WBC, or developing cold/flu).

### Randomization and Group Allocation

The participants were randomly allocated and exposed to cryogenic temperatures −110°C till −160°C (the experimental group, EG) or to low, but not cryogenic temperatures −50°C [the control group (CG)]. Patients were assigned to the groups by the researcher not involved in assessment procedures using the block (39) randomization determined by the number of persons in the cryochamber. Both investigators and subjects remained blind. There were dropouts (n=29) during 10 WBC sessions because of different reasons like previously undiagnosed (and untreated) hypertension, omitting more than two WBC sessions, or developed medical condition (e.g., developing a cold, getting burn with boiling water at home) and dropped due to personal reasons.

**Figure 1:**
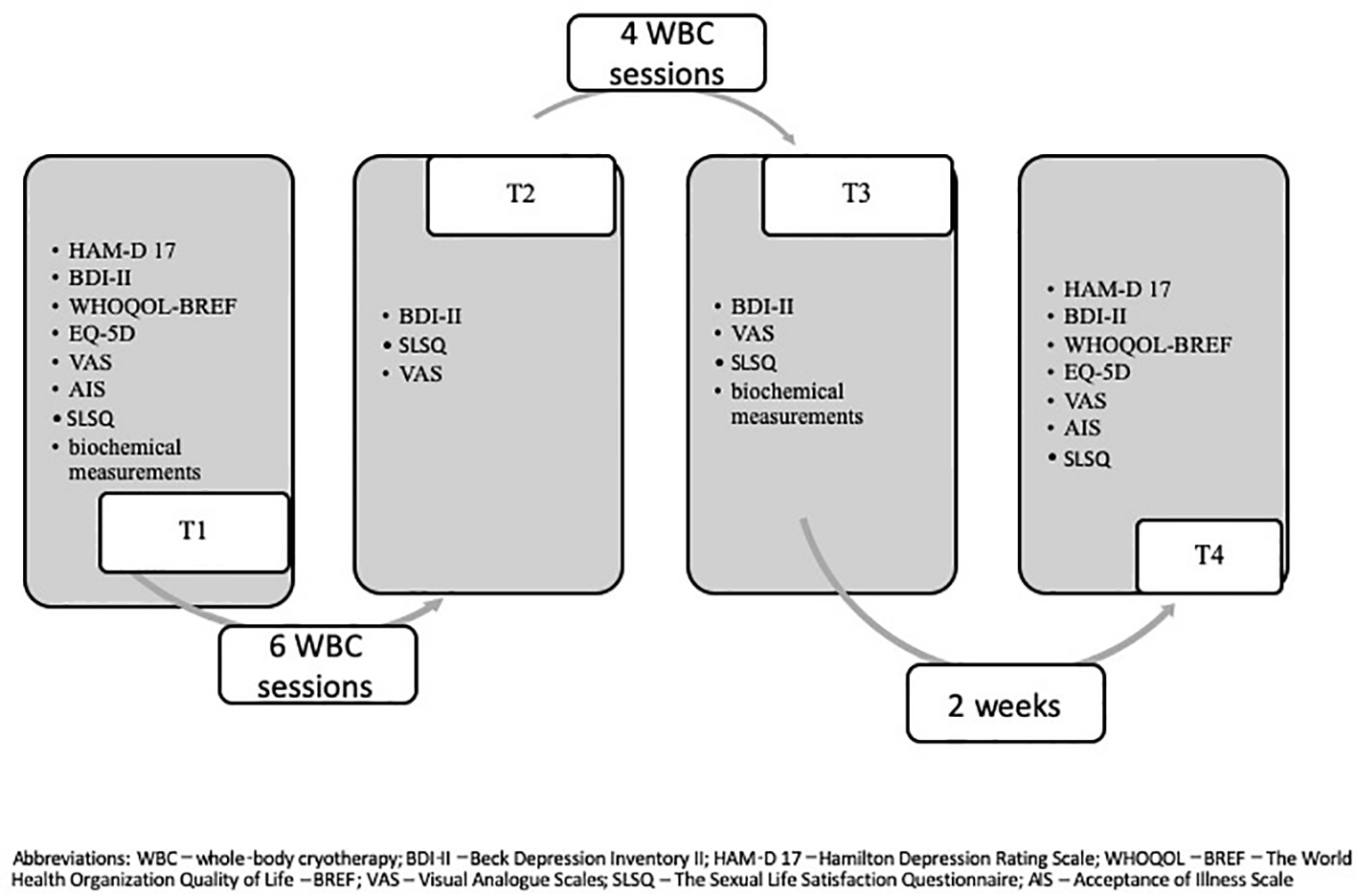
WBC depression flow diagram.

Finally, there were 30 participants (21 females, 70%) in the experimental group (mean age 46.6, range 23–73) and 26 (19 females, 73%) in the CG (mean age 48.2; range 20–69) who completed the whole study. 60% (n=30) of those who started (n=50) the study in the experimental group completed it to T4. 55% was the rate for CG (T1, n=47; T4, n= 26). Overall, the rate of people who completed the study was 58%. **Figure 1** presents the recruitment process. Moreover, 54 participants were under antidepressant (SSRI or SNRI) treatment for at least 8 weeks before WBC exposure. Only two of them were under antidepressant treatment for at least 4 weeks.

### Procedure

A cryotherapy chamber (CR 2002, Wroclaw type, provided by Creator Sp. z o. o.) was cooled by liquid nitrogen. It consisted of two rooms: the vestibule/antechamber (temperature −60°C) and the proper chamber (temperature −110°C on the first days till −135°C further on). There is no direct contact with the liquid nitrogen in this type of device.

The typical WBC session lasts for 2 min in the main chamber and is preceded by the 30 s of adaptation in the vestibule before and after the proper session. Each procedure is under control by the personnel. The group of 5–6 participants usually use it at the same time and are required to wear minimal clothing (like shorts and t-shirt), gloves, headband (or beanie), a nose and mouth mask, high-knee socks and dry shoes (e.g., wooden). This outfit should be woolen or cotton to reduce the risk of injuries caused by cold.

**Figure 2:**
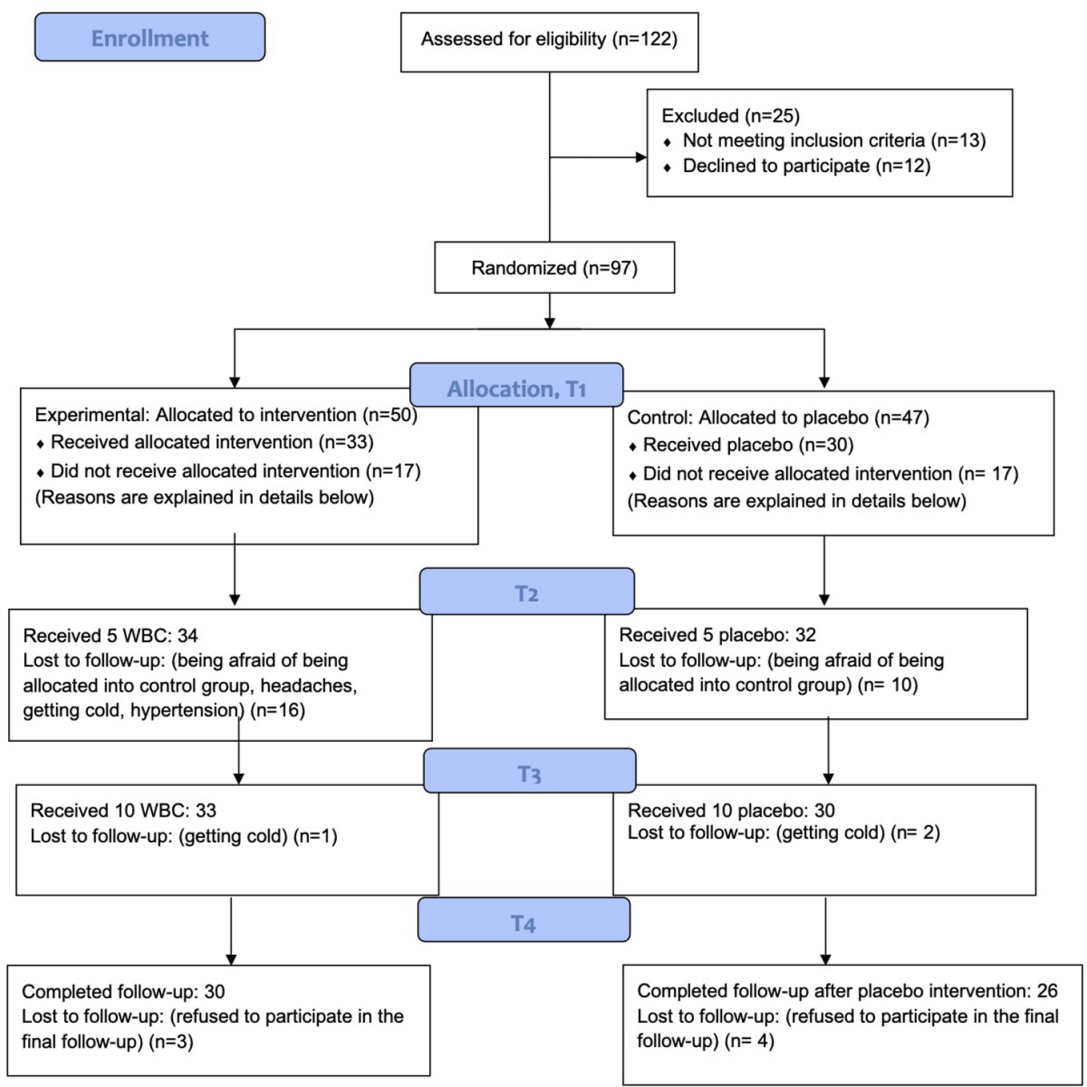
WBC study design.

Participants from the experimental group undertook 10 WBC sessions for 2 weeks (Monday–Friday, without weekends). Those from the control (sham) group undertook 10 sessions for 2 weeks (Monday–Friday) in the low temperatures −50°C but not cryogenic ones. There were four assessment visits during the study—before the first WBC session (T1), after the 6^th^ session (T2), after the last, 10^th^ WBC, (T3) and finally 2 weeks later at follow-up (T4). Psychiatric evaluations were conducted at T1 and T4. A blood examination was done at T1 and T3. **Figure 2** presents the detailed structure of the procedure.

### Measures

#### Primary Outcome Measures

The primary outcome measures were depressive symptoms evaluated with the Beck Depression Inventory-II (BDI-II) (40) and the HAM-D 17 (41). They were in details: 1) the BDI-II total score; 2) the BDI-II two subscales scores; 3) BDI-II response rates (defined as a 50% decrease in BDI-II total score as well as subscales from baseline); and 4) a depression severity evaluated by a psychiatrist HAM-D 17 before WBC and after 2 weeks from the last WBC session was also considered as a primary outcome measure. Response rated in BDI-II were rated at baseline before the first (T1), after the 6^th^ (T2), 10^th^ WBC (T3) session, and at follow-up (2 weeks from the 10^th^ session (T4).

BDI-II (40) is a self-scored tool used for the evaluation of the severity of depressive symptoms. BDI-II consists of 21 items scored between 0 (lack of symptoms) and 3 points (the highest severity of the described symptom). Total scores between 0 and 11 indicate the lack of depressive symptoms, 12–26—mild depressive episode, 27–49—moderate depressive episode, and 50–63—severe depressive episode. Authors also (40) identified two dimensions within BDI-II of self-reported depression for psychiatric outpatients in general: cognitive-affective (BDI-II D1; first 13 items) and somatic dimensions (BDI-II D2; items 14–21). The Polish version of the BDI-II has good psychometric characteristics (42).

HAM-D 17 is a widely used tool to measure depressive symptoms in psychiatry. The shorter version with 17 items was used in this study. Each symptom can be scored between 0 and 4. The total score of 7–12 points is considered to be a mild depressive disorder, 13–17—moderate, 18–29—severe, and 30–52 very severe (41).

#### Secondary Outcome Measures

The quality of life, quality of sexual life, acceptance of the disease and self-reported mood, vitality, and sleep quality were assessed as secondary outcome measures*. The World Health Organization Quality of Life*–*BREF* (WHOQoL–BREF) (43) was created based on WHOQoL-100 by The World Health Organization Group as the universal tool to assess the quality of life. It is commonly used among healthy people as well as patients diagnosed with different diseases, both for clinical and scientific purposes. It consists of 26 questions. Two questions are about the individual, general quality of life, and health. Twenty-four items are gathered into four domains: physical (D1), psychological (D2), social (D3), and environmental (D4). All questions are scored between 1 and 5. The higher summary score indicates better life quality. WHOQoL-BREF was adapted into Polish by Wołowicka and Jaracz (44).

*EQ-5D* is an instrument developed by the EuroQoL Group to measure health-related QoL. It consists of five dimensions: mobility, self-care, usual activities, pain/discomfort level, anxiety/depression; and a self-rated vertical visual analog scale (VAS) (45). It was used as an additional quality of life measure.

*The Sexual Life Satisfaction Questionnaire* (SLSQ) was used to measure sexual satisfaction. It consists of 10 items, and participants had to choose one of the following: 1—strongly disagree, 2—disagree, 3—agree, and 4—strongly agree. The result of the questionnaire is a sum of all 10 answers (some answers were re-coded) (46).

Self-report VAS were used to report on “well-being” issues, including mood (VAS1), vitality (VAS2), and sleep quality (VAS3). Participants were asked to cross the vertical line (10 cm) between two extreme points (0—the worse mood and 10—the best mood).

*Acceptance of Illness Scale* (AIS) was used to measure the level of acceptance of the disease (depression). This tool is highly recommended for use in clinical trials (47, 48).

Additionally, safety measures included adverse events reported at each visit. A physical examination (including blood pressure, heart rate) was performed since baseline till T4 at each visit.

*Blood sampling and biochemical analysis*. Venous blood samples were collected before the first session (T1), and respectively after WBC (T3). Blood samples were obtained separately into two tubes, using the S-Monovette system (Sarstedt, Nümbrecht, Germany). One tube with an appropriate amount of sodium heparin anticoagulant to obtain blood plasma, and the second (5 ml) containing a clotting activator in order to obtain blood serum. Then both types of tubes were centrifuged in standard conditions, plasma, or serum, respectively were obtained. In blood serum, hsCRP was determined by the CRP High Sensitivity kit (DiaSys, Germany), and TAS by TAS Randox kit (United Kingdom) using Konelab 20i (ThermoScientific) biochemical analyzer. The cytokine levels and NO levels were determined in blood plasma using commercially available enzyme-linked immunosorbent assay (ELISA) and the Griess method, respectively.

### Statistical Analysis

The difference between the experimental and the CG in T1 was tested using the Mann-Whitney test (for continuous variables) or Fisher’s exact test (for nominal and ordinal variables). The analysis of changes in variables in T2, T3, and T4 was done using generalized mixed models (for continuous outcome variables) or cumulative link mixed models (for ordinal variables). The frequency rate of HAM-D 17 and BDI-II was assessed using Fisher’s exact test. The analysis was performed in R for Windows (Version 3.5.3) (49). Differences were considered statistically significant if the p-value was less or equal than 0.05.

## Results

We did not observe any episodes of treatment-emergent adverse events among participants, who were carefully monitored. Every day, before each entrance to the cryochamber, participants were examined by the physician, including the assessment of blood pressure. There were no serious somatic or adverse mental events. Two people had localized skin redness on their legs, one person continued the WBC after medical consultation, and the other one decided to withdraw from the study.

### Demographic Characteristics

Fifty-six people diagnosed with depression completed the study—30 in the experimental and 26 in the CG. The mean age for the experimental group was 46.57(± 14.87) and 48.23(± 16.34) for the CG. Sixty-seven percent of people in the experimental group were married and 39% in the CG. The majority of participants were female—70% in the experimental group and 73% in the CG. Ninety-six percent of participants in both groups (96%) completed higher or secondary education. At least half of the participants had children (50% in the CG and 61% in the experimental group). The majority of people enrolled in the study lived in a big city (78% in EG and 96% in CG). People from EG statistically significantly differed from people from CG in their employment status (p=0.03). Seventy-seven percent of people from EG were employed, whereas only 39% of those from CG.

The mean time since receiving first depression diagnosis was 5.95(± 7.53) for EG and 5.38(± 7.32) for CG. In both groups, the majority of people were never admitted for hospital treatment (96% in EG and 81% in CG). **Table 1** presents detailed demographical characteristics.

**Table 1:** Demographical description of the study participants (n=56).

Variable		EG		CG		p-value
		N=30	%	N=26	%	
**Gender**	Female	21	70%	19	73%	1
	Male	9	30%	7	27%	
**Education**	Higher education	20	71%	12	52%	0.37
	Secondary education	7	25%	10	44%	
	Vocational education	1	4%	1	4%	
**Marital status**	Married	18	67%	9	39%	0.09
	Widowed	0	0%	1	4%	
	Single	9	33%	13	57%	
**Employment status**	Employed	23	77%	9	39%	0.03*
	Unemployed	1	3%	3	13%	
	Retired	5	17%	8	35%	
	Student	1	3%	3	13%	
**Children**	Yes	17	61%	12	50%	0.6
	No	11	39%	12	50%	
**City/village**	Big city	22	78%	22	96%	0.07
	City	5	18%	0	0%	
	village	1	4%	1	4%	
**Number** **of hospital treatments**	0	25	96%	13	81%	0.3
	1	0	0%	2	13%	
	2	1	4%	1	6%
	**Mean (± SD)**		**Median (Q_1_–Q_3_)**	**Mean (± SD)**	**Median (Q_1_–Q_3_)**	**p-value**
**Age**	46.57 (± 14.87)		49.5 (34–57.25)	48.23(± 16.34)	50.5(32.25–61)	0.6
**Years since** **1^st^ diagnosis**	5.95(± 7.53)		2(1–8.5)	5.38(± 7.32)	2(1–5.5)	0.9

*p ≤ 0.05.

EG, experimental group; CG, control group.

Participants were obtaining pharmacological treatment while they were participating in this RCT. **Table 2** presents detailed characteristics of prescribed medication.

**Table 2:** Baseline characteristics of pharmacological treatment (n=56).

Pharmacological Treatment		EG N=30	CG N=26
**SNRI**	Venlafaxine	11	5
	Duloxetine	1	2
**SSRI**	Sertraline	3	0
	Fluoxetine	4	3
	Citalopram	3	0
	Escitalopram	3	5
	Paroxetine	2	3
**Other groups**	Mirtazapine	0	2
	Mianserin	1	2
	Agomelatine	2	0
	Vortioxetine	0	2
	Moclobemide	0	2

EG, experimental group; CG, control group; SNRI, serotonin-norepinephrine reuptake inhibitor; SSRI, selective serotonin reuptake inhibitor.

### Level of Depressive Symptoms

Participants from the experimental group scored 16.862(± 3.796) in HAM-D 17 and 21.733(± 5.729) in BDI-II at baseline and from CG 17.346(± 6.292) and 24(± 9.499) respectively. The mean levels for BDI cognitive-affective dimension were 17.00(± 5.705) for EG and 15.385(± 6.561) for CG and somatic dimension 9.067(± 2.803) and 8.615(± 3.950) respectively. The groups did not differ statistically significantly in the level of depressive symptoms. The detailed scores are described in **Table 3**.

**Table 3:** Obtained results T1, T2, T3, and T4.

Tool	Group	T1			T2		T3		T4	
		Mean (SD)	Median (Q_1_–Q_3_)	*p*	Mean (SD)	Median (Q_1_–Q_3_)	Mean (SD)	Median (Q_1_–Q_3_)	Mean (SD)	Median (Q_1_–Q_3_)
**HAM-D 17**	EG	17.429(3.693)	17(16–19)	1	–	–	–	–	5.407(4.335)	5(2–8.5)
	CG	18.429(6.408)	19(13–23		–	–	–	–	10.571(6.250)	22(9–14)
**BDI-II**	EG	21.733(5.729)	23(18–25)	0.87	9.444(4.291)	9(6.5–12)	15.222(9.411)	15(7–22)	13.481(10.086)	13(4–21)
	CG	24.000(9.499)	21.5 (16–30.5)		18.684(9.286)	17(11–26)	18.105(10.964)	20(9–22)	20.474(11.913)	18(13–31.5)
**BDI-II D1**	EG	17.00(5.705)	18.5(13.25–20)	0.12	11.444(6.135)	11(6–16.5)	9.926(6.545)	9(4–15.5)	9.037(7.388)	9(2.5–13.5)
	CG	15.385(6.561)	14 (12–17.75)		11.947(5.949)	12(6.5–16.5)	12.105(7.497)	12(6–15.5)	13.789(8.6)	13(6.5–22)
**BDI-II D2**	EG	9.067(2.803)	9(7–11.75)	0.45	6.222(3.017)	6(4–8)	5.296(3.383)	6(2.5–8)	4.444(3.203)	5(1.5–6.5)
	CG	8.615(3.950)	8 (6.25–10.75)		6.737(3.914)	6(4–7.5)	6(4.069)	5(3–9)	6.694(3.973)	6(4–9)
**EQ-5D**	EG	7.60 (1.329)	7.5(7–8)	0.43	–	–	–	–	7.030(2.381)	6(5–8)
	CG	7.75(1.620)	8(7–9)		–	–	–	–	7.737(3.016)	8(6.5–9)
**WHOQoL D1**	EG	38.310(13.159)	38(31–44)	0.7	–	–	–	–	55.192(19.003)	56(39.5–69)
	CG	37.154(19.867)	44(20.5–48.5)		–	–	–	–	44.850(16.891)	44(31–57.75)
**WHOQoL D2**	EG	40.448(12.637)	38(31–44)	0.74	–	–	–	–	44.5(19.959)	59.5(38–69)
	CG	38.538(19.639)	38(26.5–50)		–	–	–	–	44.5(19.959)	44(31–56)
**WHOQoL D3**	EG	47.207(16.238)	50(31–56)	0.67	–	–	–	–	57.692(24.822)	62.5(45.5–69)
	CG	44.077(20.121)	44(34.25–56)		–	–	–	–	40.9*22.375)	44(31–56)
**WHOQoL D4**	EG	54.414(13.668)	50(50–63)	0.86	–	–	–	–	64.769(18.420)	63(50–79.5)
	CG	52.077(17.652)	50(44–63)		–	–	–	–	48.250(23.081)	44(31–63)
**VAS mood**	EG	3.130(1.686)	3(2–4.175)	0.69	4.903(2.308)	4.8(3.15–6.675)	5.7(2.242)	6.4(4–7.2)	–	–
	CG	3.508(1.976)	2.95(2–4.8)		4.743(1.650)	4.5(4.1–5.95)	4.952(2.022)	5(3.5–6.55)	–	–
**VAS vitality**	EG	3.467(1.624)	3.5(2.4–4)	0.67	4.953(1.892)	5(3.4–6.375)	5.56(2.059)	5.65(4–7)	–	–
	CG	3.492(2.124)	2.75(2.225–4.775)		4.996(1.814)	5(4.1–6.2)	4.965(1.94)	5.3(4.05–6.6)	–	–
**VAS sleep**	EG	3.993(2.175)	4(3–5)	0.82	4.45(2.227)	4(3–6)	5.807(2.309)	6(4.025–7.075)	–	–
	CG	3.996(2.564)	3.5(2–5.625)		5.205(2.04)	4.4(3.625–7)	5.173(2.142)	6(4.025–7.075)	–	–
**AIS**	EG	23.100(6.413)	22(18.25–27.75)	0.38	–	–	–	–	27.482(9.736)	29(22.5–35)
	CG	25.115(8.562)	25(20–29.75)		–	–	–	–	20.650(10.854)	21.5 (13.5–28.25)
**SLSQ**	EG	24.133(5.740)	24.5(21.25–27.75)	0.73	25.852(5.074)	26(23–29)	27(6.202)	27(25–30)	26.889(7.013)	26(22–32.5)
	CG	24.25(6.374)	25(20.75–28)		24.389(5.669)	25(20.75–28.50)	26.333(4.79)	26.5(25–29.75)	25.833(5.182)	25.5(24.25–27.75)

p-value between the EG and CG at T1; *p < 0.005. HAM-D 17, Hamilton Depression Rating Scale; EG, experimental group; CG, control group; BDI-II, Beck Depression Inventory-II; WHOQoL, World Health Organization Quality of Life; VAS, Visual Analog Scales; AIS, Acceptance of Illness Scale; SLSQ, The Sexual Life Satisfaction Questionnaire.

### Other Measurements

#### Quality of Life

Groups did not differ from each other in the level of quality of life. The mean levels of EQ-5D were 7.60(± 1.329) for EG and 7.75(± 1.620) for CG. The mean results in WHOQoL-BREF in EG were 38.310(± 13.159) for D1, 40.448(± 12.637) for D2, 47.207(± 16.238) for D3, and 54.414(± 13.668) for D4 and in the CG: 37.154(± 19.867), 38.538(± 19.639), 44.077(± 20.121), and 52.077(± 17.652) respectively. The mean level of life quality (EQ-5D) was 7.60(± 1.329) for EG and 7.75(± 1.620) for CG. Participants from both groups also did not differ significantly in the level of sexual satisfaction—means were 24.133(± 5.740) for EG and 24.25(± 6.374) for CG. Details are presented in **Table 3**.

#### Visual Analog Scale

Participants assessed their mood [EG: 3.130(± 1.686); CG: 3.508(± 1.976)], vitality [EG: 3.467(± 1.624); CG: 3.492(± 2.124)], and sleep quality [EG: 3.993(± 2.175); 3.996(± 2.564)] on the VAS. The groups did not differ significantly. Details are presented in **Table 3**.

#### Acceptance of Illness Scale

The experimental and the CG did not differ significantly in their level of disease acceptance. 23.1(± 6.413) was the mean in the experimental group and 25.115(± 8.562) in the CG. Detailed information is presented in **Table 3**.

### Comparison of Outcome Measures for WBC

#### Primary Outcome: Symptoms of Depression

**Table 4** presents the results of the repeated measures analysis, in which data from up to two measure points were included per participant (T1 vs. T4). The results show evidence for a statistically significant difference in the clinical assessment of depressive symptoms according to the HAM-D 17 scale (time by group interaction p=0.02). The experimental group was assessed as scored lower at the 2 weeks follow-up (T4) compared to baseline (T1), with an adjusted between-group difference in the mean score of −4.488 points. Moreover, the analysis of individual items of the HAM-D 17 scale indicates significant differences between groups measured over time in agitation (−2.69 points, p=0.03), somatic symptoms in general, (−1.86 points, p=0.05), and genital symptoms such as libido (−2.14 points, p=0.02). Our sample had sufficient power to detect changes in time (T1/T4) in the experimental group of depression severity evaluated by a psychiatrist with the HAM-D 17 and it was 99.9%.

**Table 4:** Linear mixed model analysis results (T1, T2, T3, T4).

Tool	Interaction description	*Linear mixed model analysis—interaction effect*		
		Estimate	Sth Error	*p- value*
**HAM-D 17**	(Intercept)	17.346	1.017	0.000
	T4	−7.024	1.352	0.000
	Experimental group	−0.484	1.401	0.730
	T4 time x experimental group	−4.488	1.822	0.017
**BDI-II total**	(Intercept)	24.000	1.782	0.000
	T2	−7.160	1.481	0.000
	T3	−7.944	1.501	0.000
	T4	−5.041	1.596	0.002
	Experimental group	−2.267	2.434	0.354
	T2 time x experimental group	−5.007	2.008	0.014
	T3 time x experimental group	2.010	2.023	0.322
	T4 time x experimental group	−2.939	2.126	0.169
**BDI-II D1**	(Intercept)	15.385	1.346	0.000
	T2	−4.427	0.944	0.000
	T3	−4.675	0.957	0.000
	T4	−2.712	1.018	0.009
	Experimental group	1.615	1.838	0.382
	T2 time x experimental group	−0.906	1.279	0.480
	T3 time x experimental group	−2.192	1.289	0.091
	T4 time x experimental group	−5.087	1.355	0.000
**BDI-II D2**	(Intercept)	8.615	0.706	0.000
	T2	−2.711	0.660	0.000
	T3	−3.246	0.669	0.000
	T4	−2.341	0.711	0.001
	Experimental group	0.451	0.964	0.641
	T2 time x experimental group	0.011	0.896	0.990
	T3 time x experimental group	−0.154	0.902	0.865
	T4 time x experimental group	−2.106	0.948	0.028
**WHOQoL D1**	(Intercept)	37.154	3.407	0.000
	T4	7.270	3.644	0.052
	Experimental group	1.514	4.682	0.747
	T4 time x experimental group	9.670	4.870	0.053
**WHOQoL D2**	(Intercept)	38.538	3.511	0.000
	T4	5.292	3.772	0.167
	Experimental group	2.157	4.825	0.656
	T4 time x experimental group	9.527	5.041	0.065
**WHOQoL D3**	(Intercept)	44.077	4.083	0.000
	T4	−4.720	4.068	0.252
	Experimental group	3.346	5.607	0.552
	T4 time x experimental group	15.869	5.432	0.005
**WHOQoL D4**	(Intercept)	48.692	3.400	0.000
	T4	4.205	2.482	0.096
	Experimental group	9.122	4.418	0.044
**EQ-5D**	(Intercept)	7.750	0.409	0.000
	T4	−0.037	0.571	0.948
	Experimental group	−0.150	0.559	0.789
	T4 time x experimental group	−0.509	0.757	0.504
**AIS**	(Intercept)	25.115	1.732	0.000
	T4	−3.958	2.219	0.080
	Experimental group	−2.015	2.366	0.397
	T4 time x experimental group	8.383	2.958	0.007
**VAS 1**	(Intercept)	3.508	0.399	0.000
	T2	1.321	0.351	0.000
	T3	1.539	0.361	0.000
	Experimental group	−0.378	0.545	0.490
	T2 time x experimental group	0.452	0.475	0.344
	T3 time x experimental group	1.031	0.483	0.035
**VAS 2**	(Intercept)	3.492	0.375	0.000
	T2	1.604	0.328	0.000
	T3	1.534	0.338	0.000
	Experimental group	−0.026	0.513	0.960
	T2 time x experimental group	−0.117	0.445	0.793
	T3 time x experimental group	0.559	0.453	0.219
**VAS 3**	(Intercept)	3.996	0.445	0.000
	T2	1.427	0.426	0.001
	T3	1.399	0.445	0.002
	Experimental group	−0.003	0.607	0.996
	T2 time x experimental group	−0.970	0.578	0.096
	T3 time x experimental group	0.414	0.592	0.486
**SLSQ**	(Intercept)	24.236	1.159	0.000
	T2	0.084	0.899	0.926
	T3	1.237	0.913	0.177
	T4	1.216	0.998	0.225
	Experimental group	−0.103	1.563	0.948
	T2 time x experimental group	1.383	1.210	0.255
	T3 time x experimental group	1.296	1.220	0.290
	T4 time x experimental group	1.397	1.303	0.286

HAM-D 17, Hamilton Depression Rating Scale; BDI-II, Beck Depression Inventory-II; WHOQoL, World Health Organization Quality of Life; VAS, Visual Analog Scales; AIS, Acceptance of Illness Scale; SLSQ, The Sexual Life Satisfaction Questionnaire.

The percentage of participants who were assessed by a clinician as scored less than 8 (that is, “recovered”) was 66.7% in the experimental group at 2 weeks follow-up (p=0.55; compared with 57.14% in the CG).

**Table 4** illustrates the results of the repeated measures analysis, in which results from up to four data collection points were included per participant. According to the total score in BDI-II, there was evidence of a difference between the EG and the CG between baseline (T1) and after 1 week of WBC (T2) (time by group interaction p=0.01) reported by subjects themselves, with an adjusted between-group difference in the mean score of −5.007 points. The BDI-II differences were not maintained throughout time. The results obtained in T3 and T4 showed no significant differences between groups. Nevertheless, according to the subjects, some areas that were related to the great influence of WBC also in the last assessment (T4) after 2 weeks of the last WBC session (T4) and differed significantly compared to the CG. They were sadness (item 1; p=0.002), loss of pleasure (item 4; p=0.02), self-criticalness (item 8; p=0.045), crying (item 10; p=0.033), loss of interest (item 12; p=0.013), loss of interest (item 12; p=0.013), and indecisiveness (item 14; p=0.025). The statistical power of detecting the changes in time (T1/T2/T3/T4) in the experimental group in depressive symptoms evaluated with the BDI-II was 56.4%, which indicated medium power.

The percentage of participants who reported scores less than 10 (that is, “recovered”) was 73.33% in the experimental group at 2 weeks follow-up (p=0.00; compared with 28% in the control arm).

Significantly important changes were noticed for cognitive-affective and somatic dimensions in BDI-II. Participants from the experimental group scored significantly less in the cognitive-affective dimension (time by group interaction p=0.00) comparing to the CG at T4 (2 weeks after the last WBC session). The same result was observed for the somatic dimension (p=0.028).

#### Secondary Outcomes

##### Quality of Life

Significant improvement was noticed in life quality among participants from the experimental group comparing to the CG. It is related to social (D3) (p=0.005) and environmental (D4) (p=0.044) dimensions between T1 and T4—2 weeks after the last WBC session. Improvement was not noticed in D1 and D2, as well as in the general quality of life measured with the EQ-5D scale. However, changes were noticed for EQ-5D self-rated health on the VAS (p=0.042), in the level of pain/discomfort (p=0.003), and self-assessed level of anxiety/depression (0.021). There were no statistically significant changes related to sexual satisfaction (p > 0.05). All details are presented in **Table 4**.

##### Self-Assessed Mood, Vitality, and Sleep Quality

Participants from EG also reported significant improvement (T3 time and group p=0.035) in their mood comparing to those from CG using VAS. These changes were not noted for self-assessed vitality and sleep quality. **Table 4** presents detailed information.

##### Acceptance of Illness Scale

Also, the positive changes were noticed relating to the level of disease acceptation (p=0.007) between baseline and 2 weeks after the last WBC session compared to the CG. **Table 4** presents detailed information.

### Inflammatory and Antioxidant Status’ Assessment

A comparison of blood biochemical parameters assessed in both the experimental and the CGs before (T1) and after 2 weeks of WBC (T3) (see **Figure 1**) are presented in **Table 5**. There were no significant differences between-collection time and between-groups in hs-CRP, IL-6, IL-10, NO, and antioxidative status–index (p > 0.05).

**Table 5:** Biochemical results in blood before and after WBC (T1 vs. T3): hsCRP, TAS, IL-6, IL-10, and NO in experimental and CGs.

Parameter	Group	T1 Median (Q1–Q3) Mean ± SD	T3 Median (Q1–Q3) Mean ± SD	Interaction description	*Linear mixed model analysis—interaction effect*		
					Estimate	Sth Error	*p- value*
**hsCRP [mg/L]**	EG	1.090 (0.580–3.133) 2.105 ± 2.135	1.179 (0.605–3.442) 2.689 ± 3.115	(Intercept) T3 Experimental group	2.232 0.339 −0.005	0.459 0.413 0.554	0.000 0.415 0.993
	CG	1.900 (0.400–4.240) 2.387 ± 2.234	1.179 (0.670–3.312) 2.431 ± 2.658				
**IL-6 [pg/ml]**	EG	0.000 (0.000–6.860) 26.955 ± 94.241	0.000 (0.000–8.625) 29.924 ± 111.861	(Intercept) T3 Experimental group	5.532 1.570 22.122	14.604 1.968 21.329	0.707 0.429 0.305
	CG	0.000 (0.000–7.107) 6.144 ± 16.940	0.000 (0.000–5.234) 6.491 ± 18.046				
**IL-10 [pg/ml]**	EG	5.686 (3.732–7.130) 6.360 ± 5.390	5.686 (5.009–7.130) 6.951 ±4.699	(Intercept) T3 Experimental group	7.518 0.874 −1.300	1.165 0.465 1.653	0.000 0.067 0.436
	CG	6.535 (4.427–8.282) 7.389 ± 5.060	6.350 (4.487–10.562) 8.522 ± 7.184				
**TAS [mmol/L]**	EG	1.520 (1.470–1.630) 1.537 ± 0.135	1.520 (1.443–1.630) 1.539 ± 0.141	(Intercept) T3 Experimental group	1.501 0.020 0.027	0.025 0.022 0.030	0.000 0.366 0.377
	CG	1.470 (1.410–1.600) 1.492 ± 0.127	1.520 (1.440–1.625) 1.531 ± 0.149				
**NO [nM]**	EG	459.973 (283.504–676.548) 516.739 ±371.622	524.143 (157.169–810.905) 865.864 ±1434.050	(Intercept) T3 Experimental group	2033.609 −64.892 -−129.570	760.832 130.334 928.640	0.015 0.624 0.239
	CG	315.589 (143.131–2626.274) 2087.044 ±3266.396	652.484 (427.887–2296.242) 1915.282 ±2575.159				

EG, experimental group; CG, control group; TAS, total antioxidant status; NO, nitric oxide; hsCRP, High sensitivity C-reactive protein; IL, Interleukin.

## Discussion

This study highlighted the effectiveness of 10 sessions of cryotherapy (WBC) in reducing the symptoms of mood disorders in patients with the diagnosis of depression. Study participants observed the positive effect after the first week of this biological intervention, while in a clinical assessment, the effectiveness was visible after 2 weeks of the end of WBC. Moreover, from the participant’s perspective, the improvement was maintained throughout time in psychopathological symptoms of depression such as sadness, loss of pleasure, self-criticalness, crying, loss of interest, loss of interest, and indecisiveness (T1 vs. T4). Furthermore, in the psychiatric evaluation, clinicians noticed improvements primarily in agitation, somatic symptoms in general, and libido. Additionally, the present study showed that WBC intervention could be beneficial for well-being and some aspects of quality of life. The positive changes were noticed relating to the mood and level of disease acceptance, but no significant changes were observed in sleep quality and sexual satisfaction. It was also shown that WBC-dependent mental health improvement is not connected with the down-regulation of the inflammatory response.

According to the current state of knowledge, practical applications of a whole- or partial-body cryotherapy can be observed, especially in rheumatoid arthritis, but also in ankylosing spondylitis, fibromyalgia and multiple sclerosis, and chronic low back pain [for review see: (14)]. To date, only several studies have aimed to address the alternations of mental health after WBC exposition (19–21, 50, 51). In our study, results imply that 10 sessions of WBC beneficially reduce depressive symptoms, including somatic domains; however, with a short-lived effect (T1 vs. T2).

Indeed, our present findings confirm the results of earlier studies of Rymaszewska et al. (19, 20), and also our preliminary report (21). In 2012, Misiak et al. (52) suggested translation WBC into the prevention of MCI and Alzheimer’s disease. Furthermore, our group confirms this hypothesis in MCI patients, where the improvement in cognitive functioning was observed (22). These participants did not suffer from depression. Nevertheless, they declared more energy and vitality after WBC, which is consistent with these findings. Some evidence showed that depression in later life is associated with increased risk of cognitive impairment, which can often be developed after the onset of mood symptoms [for review see: (53, 54)]. Therefore, these findings encourage implementation of the WBC as a preventive intervention in late-life depression, especially linked with cognitive decline.

In our study, we also have shown that WBC significantly improved some aspects of the quality of life of experimental group patients compared to control. It is related to social and environmental dimensions between T1 and T4—2 weeks after the last WBC session (HAMD-17 and BDI-II scales). These findings are consistent with data from previous studies of Rymaszewska’s group (51), showing the effectiveness of cryotherapy in patients with spinal pain- and peripheral joint disease. Potential mechanisms of action were probably connected with the pain relief after WBC. In our case, the reduction of depressive symptoms seems to be crucial concerning the self-reported quality of life improvement. We also saw that participants from EG subjected to 10 sessions of WBC had reported significant improvement in their mood compared to those from CG. Also, the positive changes were noticed relating to the level of disease acceptance. Moreover, it has to be noted that exposition to extremely low temperatures may improve libido and sleep quality, which is strongly connected with the quality of life. However, we do not observe any changes in reported sexual satisfaction. We hypothesize that this fact may be due to the very short period of follow-up assessment. Douzi et al. (55) revealed that 3-min WBC (also called “cryostimulation”) after training improves subjective and objective sleep quality in physically active men. Interestingly, we did not find improvement in the self-reported sleep quality. It should be noted that our participants do not attend to exercise training before or after WBC. Moreover, exercise training during the evening may influence sleep conditions (55).

WBC might be an effective and safe intervention in patients with depressive disorders (21). Nevertheless, the exact mechanisms explaining the beneficial effect of WBC on mood disorders remain unresolved. One of the potential mechanisms is linked to modulating inflammatory response. Some previously published studies revealed that WBC could be useful to limit the inflammatory process (14, 56). Mostly, the assessment of the occurred inflammation was connected with physical activity. For example, it has been revealed that WBC may influence the increase of anti-inflammatory cytokines, such as IL-6 and IL-10, (57–60) and decreased pro-inflammatory cytokine IL-1β (58). Additionally, hsCRP was decreased in male ankylosing spondylitis patients, (26) and in middle-aged, obese men (61), while in athletes was unchanged (62). Indeed, we previously observed the reduction of IL-6 in MCI patients and the release of NO, which is an important mechanism controlling vasomotor function (22). These findings suggested that cryotherapy influences on immunological status. Nevertheless, the results in this study confirmed our preliminary report (21) that biochemical parameters in both the experimental and the CGs remain unchanged. We did not observe any significant changes in the level of interleukin 6, interleukin 10, and hsCRP level. Trying to explain these observations should take into account the fact that, during WBC, the patients were under standard antidepressant treatment. The meta-analysis of Więdłocha et al. (63) revealed that after antidepressant drugs, the level of IL-4, IL-6, and IL-10 were decreased in patients with major depressive disorder. As we previously speculated (21), the IL-6 plays an important role in the production of hsCRP in the liver, and lack of changes in IL-6’ level might be connected with no changes in hsCRP levels. This issue is the convincing evidence that conducting such a study on antidepressant-naive patient could be eligible, which is obviously, in our opinion, ethically controversial. Thus, the biochemical results call for caution when discussing findings.

Mood disorders might also be linked with oxidative and nitrosative stress (61). Recently published reports suggested the increase of the activity of antioxidant enzymes (64) and the beneficial effect on other oxidative stress markers in rats (65). The decrease of parameters of oxidative stress or increase of total antioxidative status in healthy men and depressive multiple sclerosis patients (66, 67) were also observed. These encouraged us to explore the level of NO and total antioxidative status. Unfortunately, no differences were found in the level of parameters tested in WBC patients compared to the CG. It might be assumed that antidepressant treatment is associated with these results, for as much it has been revealed that SSRI implementation may affect the oxidative stress status and NO likewise (68, 69).

The results of our study should be interpreted after considering several limitations. Firstly, the study sample, which completed the study and was considered in the statistical analysis, was relatively small. Especially, comparing to the number of recruited participants to the study. This makes it less reliable for the multivariate analyses. However, the multivariate analyses method was chosen by the researchers as it allowed the more complex insight into the obtained results. It would not be possible with use of multiple simple statistical tests. Participants did not receive any financial or material gratification for participation in the study. They were supposed to participate in at least eight of 10 WBC sessions to complete the study and in the follow-up meeting after 2 weeks. Detailed information about the numbers of participants who completed the following study steps is included in **Figure 2**. Secondly, 54 participants were under antidepressant (SSRI or SNRI) treatment for at least 8 weeks before WBC exposure. Only two participants were under antidepressant treatment for at least 4 weeks. It has to be pointed out that the CG patients with depression who did not undergo cryogenic temperature, but received standard treatment. Obviously, WBC was provided as an adjuvant treatment that can improve pharmacological treatment. It was consistent in the line of our hypothesis. Nevertheless, antidepressants may influence the inflammation process, and this issue is difficult to be excluded in studies of patients with various stages of depressive disorder. Additionally, the majority of participants were female. As we recently discussed (21), it might influence the cytokines index. It should also be noted that 2 weeks of cryotherapy may be profitable to health, for example, through the displacement of loneliness or lack of any social activity. WBC sessions conduce to social contacts with other patients. Although in our study, we controlled the effect of WBC after 2 weeks since last session, the prolonged follow-up examination is needed to determine how long the beneficial WBC intervention is maintained. Moreover, the extending of the WBC from 10 standard sessions could be pivotal and may influence the obtained effect.

## Conclusions

In summary, findings from our study point to the conclusion that WBC is a useful method to improve standard pharmacological treatment. The WBC intervention reduces mental health deterioration, especially in mood disorders, such as depression, and can be beneficial for well-being and quality of life. Moreover, WBC appears to be safe in standard 10-session implementation. Nevertheless, future research should look more carefully into the underlying biological mechanisms, instead of focusing solely on relationships between WBC and psychopathological assessment in mental disorders.

## Data Availability Statement

The datasets generated for this study are available on request to the corresponding author.

## Ethics Statement

The project was based on prospective randomized, double-blind sham-controlled protocol reviewed and accepted by the Bioethical Committee (permissions numbers: KB-406/2014 and KB-252/2015) and was conducted in accordance with the principles of Good Clinical Practice and the Declaration of Helsinki.

## Author Contributions

Study design: BS, DS, and JR. Recruitment and assessment of psychopathology: JR, KL, BS, TP, and ET. Blood parameters analysis: LP-S and AZ. Other data analysis: KL, DS, BS, JER, and JR. Manuscript writing: JR, KL, DS, AZ, and BS. All authors have approved the final manuscript.

## Funding

The study was evaluated and funded by the Ministry of Science and Higher Education (registration number Pbmn 167), awarded to BS, within the framework of research aimed at promoting young scientists in cooperation with the company Creator Sp. z o.o. in Wrocław. The funding institution had no role in the study design, collection, analysis or interpretation of the data, writing the manuscript, or the decision to submit the paper for publication.

## Conflict of Interest

The authors declare that the research was conducted in the absence of any commercial or financial relationships that could be construed as a potential conflict of interest.

## Abbreviations

WBC, whole-body cryotherapy; BDI-II, Beck Depression Inventory-II; HAM-D 17, Hamilton Depression Rating Scale; EG, experimental group; CG, control group; rTMS, repetitive transcranial magnetic stimulation; PBMC, peripheral blood mononuclear cells; TAS, total antioxidant status; WHOQoL, BREF – The World Health Organization Quality of Life – BREF; VAS, Visual Analog Scales; SLSQ, The Sexual Life Satisfaction Questionnaire; AIS, Acceptance of Illness Scale; NO, nitric oxide; SSRI, Selective Serotonin Reuptake Inhibitor; SNRI, Serotonin-Norepinephrine Reuptake Inhibitor.
